# Parsley: a web app for parsing data from plate readers

**DOI:** 10.1093/bioinformatics/btad733

**Published:** 2023-12-04

**Authors:** Eszter Csibra, Guy-Bart Stan

**Affiliations:** Department of Bioengineering, Imperial College Centre for Synthetic Biology (IC-CSynB), Imperial College London, London SW7 2AY, United Kingdom; Department of Bioengineering, Imperial College Centre for Synthetic Biology (IC-CSynB), Imperial College London, London SW7 2AY, United Kingdom

## Abstract

**Summary:**

As demand for the automation of biological assays has increased over recent years, the range of measurement types implemented by multiwell plate readers has broadened and the list of published software packages that caters to their analysis has grown. However, most plate readers export data in esoteric formats with little or no metadata, while most analytical software packages are built to work with tidy data accompanied by associated metadata. ‘Parser’ functions are therefore required to prepare raw data for analysis. Such functions are instrument- and data type-specific, and to date, no generic tool exists that can parse data from multiple data types or multiple plate readers, despite the potential for such a tool to speed up access to analysed data and remove an important barrier for less confident coders. We have developed the interactive web application, Parsley, to bridge this gap. Unlike conventional programmatic parser functions, Parsley makes few assumptions about exported data, instead employing user inputs to identify and extract data from data files. In doing so, it is designed to enable any user to parse plate reader data and can handle a wide variety of instruments (10+) and data types (53+). Parsley is freely available via a web interface, enabling access to its unique plate reader data parsing functionality, without the need to install software or write code.

**Availability and implementation:**

The Parsley web application can be accessed at: https://gbstan.shinyapps.io/parsleyapp/. The source code is available at: https://github.com/ec363/parsleyapp and is archived on Zenodo: https://zenodo.org/records/10011752.

## 1 Introduction

Most modern plate readers are capable of carrying out absorbance, fluorescence and luminescence readings using multiwell plates ranging from 6- to 384-well plates (with 96-well plates being the most common). Many come with additional features to enable spectrum scanning, timecourse measurements, as well as the ability to control temperature, tune wavelengths or implement reagent injection. As a result of this versatility, a diverse range of phenotypes may be assayed in these instruments in high-throughput, making plate readers essential tools for the average molecular or cell biology laboratory.

A growing number of analytical packages have been developed in recent years to automate the analysis of plate reader data. A list of currently available packages is shown in Supplementary Table S1 (Fernandez-Ricaud *et al.* 2016, Sprouffske and Wagner 2016, Coutin 2019a, Fernandez 2019, Martin *et al.* 2019, Coutin *et al.* 2020, Fedorec *et al.* 2020, Kamrad *et al.* 2020, Small *et al.* 2020, Sprouffske 2020, Cheng and Thrash 2021, 2023, Csibra 2021, Kamrad 2021, Midani *et al.* 2021, Shterev *et al.* 2021, Warchal 2021, Wirth 2021, Yáñez Feliú *et al.* 2021, Csibra and Stan 2022, Montaño-Gutierrez *et al.* 2022, Osthege 2022, Osthege *et al.* 2022, Small and McCormick 2023, Swain 2023, Blazanin 2023a, b, Fedorec 2023, Giordano 2023, González-Cebrián *et al.* 2023, González-Cebrián and Vignoni 2023, Midani 2023). These tools fall broadly into three categories: those that tackle the analysis of microbial growth curves, those that quantify fluorescent protein expression, and those that evaluate phenotype (or drug) screening assays. Regardless of type, all analysis workflows must begin with the importing and cleaning of raw data. Raw data files produced by plate readers tend to be exported in Microsoft Excel format, but they suffer from a lack of data standardization [unlike e.g. standardized FCS file formats exported by flow cytometers (Spidlen *et al.* 2021)]. This means that the exact location of the data within the file, the orientation and arrangement of the data, and the presence and location of metadata all depend on the commercial software sold with the instrument. However, analytical functions rely on data (i) in so-called ‘tidy’ format [where every column is a variable, every row is an observation, and every cell contains a single value (Wickham 2014)] and (ii) which includes all the necessary metadata for the downstream analysis. The conversion of raw data into a format appropriate for programmatic analysis—the combination of data extraction, data tidying and metadata joining known as data ‘parsing’—is therefore an essential primary requirement for the analysis of plate reader data.

As shown in Supplementary Table S1, some tools expect raw plate reader data as inputs. As raw data export formats differ between instruments, these packages restrict their user base to only those who own identical plate readers (or those who are willing to edit package functions). In contrast, packages that require tidy data inputs for their analytical functions, enable the modularization of their workflow by separating the data parsing from the data analysis. Parser functions can then be supplied as needed by either the package authors or external sources without affecting the downstream analysis.

Data parsing functions can be found in a variety of packages [Supplementary Table S2 (Fernandez-Ricaud *et al.* 2016, Hughes 2016, 2022, Coutin 2019a, b, c, d, e, Fernandez 2019, Martin *et al.* 2019, Coutin *et al.* 2020, Fedorec *et al.* 2020, Palakkan 2020, Palakkan and Davies 2020, Vegh 2020, Csibra 2021, Mante 2021, Shterev *et al.* 2021, Wirth 2021, Csibra and Stan 2022, Montaño-Gutierrez *et al.* 2022, Osthege 2022, Osthege *et al.* 2022, Blazanin 2023a, b, Fedorec 2023, Giordano 2023, González-Cebrián and Vignoni 2023, González-Cebrián *et al.* 2023, Midani 2023, Samineni *et al.* 2023, Swain 2023)]. Of the 22 tools identified, parsers in 15 of these function in data extraction, 16 in data tidying and 11 in metadata joining, though capabilities in each area vary. For instance, the data extraction capabilities of many of these tools are limited to only using the simplest data types (i.e. not timecourse data), by only one data format (e.g. can only extract data from column formatted files), or where only 1 reading has been taken during an experiment. Most of these tools are analysis packages that contain the occasional parsing function, but a few tools were written specifically as parsing software: AUDIT, plater, PLATEO and XDC (Hughes 2016, 2022, Coutin 2019a, Coutin *et al.* 2020, Vegh 2020, Mante 2021, Samineni *et al.* 2023). For instance, the widely used plater package was developed specifically to automate the joining of data and metadata (Hughes 2016, 2022). However, plater (i) requires metadata input to be provided in 8 by 12 matrix format, which can be impractical when a large number of metadata variables need to be recorded, and (ii) assumes that the data itself is already in tidy format. Most include parsers for only one or two instruments, while bletl, FLAPJACK, flopr, AUDIT, PLATEO and QurvE include parsers for (certain export formats from) 3, 3, 3, 4, 6 and 8 instruments, respectively (Coutin 2019a, Coutin *et al.* 2020, Fedorec *et al.* 2020, Vegh 2020, Wirth 2021, Yáñez Feliú *et al.* 2021, Fedorec 2023). While gcplyr can handle data extraction from row-, column- and matrix-formats, it is built for microbial growth curve data, and does not consider other data types (Blazanin 2023a, b).

Thus, none of the above tools tackle the generic problem of data extraction from diverse raw data structures, nor are they implemented as interactive parsing applications. As a result, prospective users of plate reader analysis software are likely to have to write one, or perhaps several, parser functions before being able to begin their analyses. This requirement for function writing poses a hurdle that impedes the take-up of software packages for data analysis, particularly for users who are less confident in their coding abilities.

To confront this problem, we have developed Parsley (Csibra 2023a, b), a web-based, universal plate reader data parsing application written in R/Shiny (Chang *et al.* 2022), that aims to enable users to parse raw plate reader data from any plate reader in under 5 min. As Parsley enables users to parse data without having to write any code, it is anticipated that it will reduce barriers to the use of the wide array of excellent data analysis software that is actively being developed by the research community.

## 2 Results

### 2.1 Parsley overview

**Figure 1.**
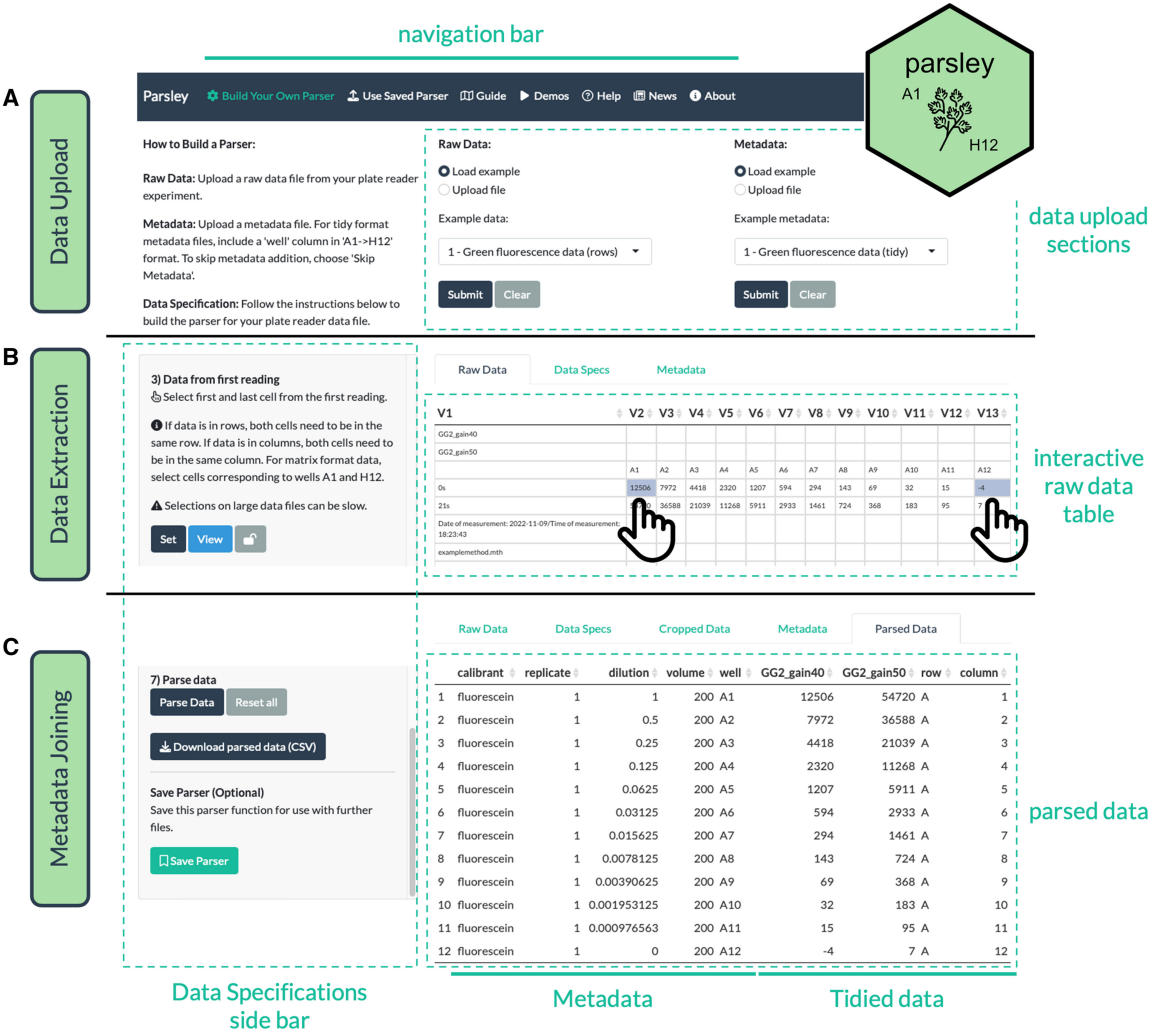
The Parsley workflow. Screenshots of the Parsley app UI at three stages. (A) Raw Data and Metadata upload sections. (B) Data Specifications step 3: selection of first and last cells for the first reading using the interactive DataTables table. (C) The final Data Specifications step 7, in which the data, having been extracted in previous steps, is tidied and joined to the provided metadata, and can be downloaded as a CSV. The built parser function can then also be saved for reuse at a later date.

There are three main steps to parsing plate reader data with Parsley (Fig. 1). First, an experimental Raw Data file needs to be uploaded (Fig. 1A). Second, an accompanying Metadata file containing all the variables required for downstream analysis needs to be created and uploaded (Fig. 1A). Parsley accepts files in a range of formats (CSV, CSV2, TSV, .txt and Excel). Third, users need to proceed through a guided series of Data Specification steps, that informs the app about key properties of the data in the Raw Data file (Fig. 1B and C). The user interface (UI) is organized with the Raw Data and Metadata upload modules at the top, and the Data Specifications options in a left-hand sidebar. To avoid overloading users with data entry fields, the UI is configured to gradually reveal options as the user interacts with the software.

### 2.2 A framework for a general data parsing application

While raw data from plate readers is exported in a wide range of formats, there are some conventions to data export formats that can be used as starting points for a general parser. For example, the most common multiwell plate is the 96-well plate, in which rows are numbered ‘A’ to ‘H’, columns ‘1’ to ’12’, and wells ‘A1’ to ‘H12’. Most export functions will present this data in one of three formats: horizontal (readings in rows, wells in columns), vertical (readings in columns, wells in rows), or matrix format (presented as a grid that visually mimics a multiwell plate), where a reading refers to a measurement (such as OD600, or green fluorescence). For horizontal and vertical data, well order is typically ‘A1’, ‘A2’, ‘A3’, etc. For matrix data, the entire plate is typically represented as an 8 by 12 grid. For horizontal/vertical data, consecutive rows/columns are typically used for each respective measurement reading. Therefore, some assumptions can be made about data structures and built into a general parser. The rare exceptions can then be handled by adapting the default framework.

### 2.3 The DT package enables data extraction parameters to be specified interactively in Shiny

Unlike a standard parser function, which may identify the location of data within a raw data file programmatically by instrument-specific context clues, Parsley identifies the location of data entirely from positional information. This is because Parsley makes few assumptions about the raw data structure and does not aim to provide automation capabilities. However, Parsley is intended to be generic to a wide range of formats. Positional information is also an easy and intuitive type of input to ask users to provide. To implement this, the app uses DataTables from the DT package (Xie *et al.* 2023) to display the Raw Data file. DT renders an interactive HTML widget that displays the Raw Data dataframe, allows for cells in the table to be interactively selected (Fig. 1B), and makes the indices of the selected cells available to the Shiny server. These can then be used to extract the data from the original Raw Data file.

### 2.4 Metadata joining

Once the data is extracted from the raw data file, it is tidied and joined to user-specified metadata. There are few requirements for the metadata file, except that it needs to contain a variable called ‘well’, as this is the column that is used for joining the data and metadata together. All other columns are optional and will depend on downstream requirements. The parse function in Parsley builds a tidy dataframe consisting of the metadata on the left and the tidied data on the right, which can be downloaded as a CSV file and used in downstream analyses (Fig. 1C).

### 2.5 Guided parsing ensures step-by-step validation and minimizes parsing errors

Parsley is built to guide users through a predefined series of steps to specify the type and format of the data, the location of the data, and the number and name of readings and/or timepoints (for timecourse data) and/or wavelengths (for spectra). A number of checks are included in both the UI (for the user to validate) and the server (for the program to validate). For instance, each Data Specification step involves a user entered action (a dropdown selection, a text entry or a cell selection on the raw data) followed by a submission with a button labelled ‘Set’. Invalid entries are flagged by an error message that informs the user about the detected data entry problem. Similarly, a Data Specifications tab collects all the user inputs, which can be checked to verify that the program is using the values that the user intends Parsley to be using. Most errors should be caught with a combination of these methods. A complete guide to the use of the app is available in the ‘Guide’ tab in the main header, with a ‘Demos’ tab containing screen recording solutions for all three data types, as well as a ‘Help’ tab to troubleshoot common issues and provide definitions for the error messages.

### 2.6 Using Parsley to build and save parser functions

In completing the above steps, users not only parse a Raw Data file, but also effectively ‘build’ their own parser functions. To speed up the parsing of similar data files, Parsley allows users to save completed parser functions as .RDS (R data) files (Fig. 1). A dedicated ‘Use Saved Parser’ tab then allows users to load previously saved parser functions at a later date, to parse further files, without requiring users to go through the Data Specification steps again. and simply requires the upload of a Parser, Raw Data and Metadata files, and clicking ‘Run Parser’.

## 3 Conclusion

Parsley tackles an as-yet-unaddressed problem in the multiwell plate data analytics pipeline, namely the question of whether it is possible to write generic data parsing software that operates on raw data exported from any plate reader instrument. Compared to other parsing tools, Parsley is designed with as few assumptions as possible about the raw data and maximum flexibility to deal with unusual data types. These options allowed us to verify that Parsley can extract data successfully from at least 53 export formats from over 10 different instruments (Supplementary Fig. S2). It is implemented as an interactive web-based Shiny app, which is key to its universal functionality as well as its accessibility to noncoders. We hope it will become a useful tool for the community and will facilitate and streamline the use of downstream plate data analysis software.

## Supplementary Material

btad733_Supplementary_Data

## Data Availability

No new data were generated or analysed in support of this research.
